# *Morchella esculenta* (L.) Pers. Wild of the Province of Taza, Morocco: Toxicity Assessment in In Vitro and In Vivo Models, Antioxidant Activities and Correlation With the Chemical Composition of Aqueous and Organic Extracts

**DOI:** 10.1155/bri/4816057

**Published:** 2025-12-17

**Authors:** Asmae Mahtal, Fatima Lamchouri, Nacima Lachkar, Hafida Bouhazama, Hamid Toufik

**Affiliations:** ^1^ MPCAE Laboratory-Materials, Natural Substances, Environment and Modeling Team (MSNEM), Polydisciplinary Faculty (FP), Sidi Mohamed Ben Abdellah University (USMBA), B.P.: 1223 Taza-Gare, Taza, Morocco

**Keywords:** antioxidant activity, *in vitro* and *in vivo* toxicity, *Morchella esculenta* (L.) pers., principal component analysis

## Abstract

Mushrooms are a source of nutrients and bioactive substances. This work, carried out for the first time, aims to evaluate in vitro and in vivo toxicity and antioxidant potential of the wild edible mushroom from the province of Taza, Morocco: *Morchella esculenta* (L.) Pers., the extraction is carried out using two methods: aqueous and organic. We assessed the toxicity in vitro on *Artemia salina* larvae and in vivo on Swiss Albino mice. Antioxidant activity was assessed by H_2_O_2_, ABTS, FRAP and PR tests. Principal component analysis enabled us to visualise the correlations between the chemical composition, antioxidant activity and in vitro toxicity. The in vitro toxicity results showed that the infused extract was nontoxic (LD50 = 3443.33 ± 20.66 μg/mL) and the diethyl ether macerated was toxic (LD50 = 68.91 ± 2.76 μg/mL). In vivo, this mushroom is not acutely toxic (LD50 ≥ 5000 mg/kg body weight). This study revealed that the decocted was the most active via the H_2_O_2_ test (23.69 ± 0.61%) and the aqueous macerated was the most active through the ABTS and FRAP tests, with 184.1 ± 0.67 mg TE/g E and 137.92 ± 0.03 mgET/gE, respectively, while the diethyl ether macerated was the most active via PR test (43.76 ± 0.51 mgEAA/gE). Principal component analysis shows a strong correlation between polyphenols and the FRAP test (*r* = 0.8369) and flavonoids and the PR test (*r* = 0.8484). There is a strong correlation between in vitro toxicity, catechic tannin content and reducing power, with *r* = 0.8079 and *r* = 0.7869, respectively. Thus, consumption of this mushroom after heat treatment is considered safe and it has an interesting antioxidant potential, which could offer it therapeutic value.

## 1. Introduction

Free radicals are elements that contain one or more free electrons, making them highly reactive [1]. They are produced in the mitochondria and cytosol in the form of superoxide ions, hydrogen peroxide and OH^−^ radicals [2] and are subsequently considered to be triggers for a number of diseases, especially those associated with ageing, such as cancer, eye diseases and neurodegenerative diseases [3]. These problems arise when there is an imbalance between the quantity of free radicals produced by cellular metabolism and antioxidants [4]; hence, this highlights the need for exogenous antioxidants such as vitamins C and E, carotenoids and phenolic compounds, which can be obtained from nutrition [5].

Almost 2000 species of macro fungi have been identified as edible species, differentiated by their fruiting bodies, which may be epigeous (aerial) or hypogeous (underground). These macro fungi are involved in vital processes in the forest ecosystem [6–10]. In addition to this role, edible mushroom plays an important role in the diet and in therapy. In fact, they are considered to be an interesting source of essential nutrients for the proper functioning of the human body (essential amino acids, fibre, minerals and others) and also a source of bioactive substances such as antioxidants, which are essentially phenolic acids and flavonoids, followed by ascorbic acid, carotenoids, ergothioneine and tocopherols. The antioxidant effect is due to their potential to neutralise oxidising agents by generating hydrogen from phenolic groups [11].

Mushroom poisoning can be serious, even fatal, due to confusion between toxic and edible mushrooms, caused by incorrect identification of the mushroom. Poisoning can also be caused by eating a mushroom contaminated either by a microbial source or by chemical substances such as pesticides [12]. The toxicity of mushrooms is linked to their toxins, such as amatoxins, phallotoxins and virotoxins, which are capable of causing abnormalities, such as toxic hepatitis [13].

The present work, carried out for the first time, aims to assess the in vitro antioxidant potential of aqueous (decocted, infused and macerated) and organic (ethanolic extract, ethanolic macerated, acetonic extract, acetonic macerated, diethyl ether extract and diethyl ether macerated) extracts of the edible mushroom *Morchella esculenta* (L.) Pers. wild from the Taza region of Morocco, using four tests (H_2_O_2_, ABTS, FRAP and PR). Since mushrooms are known for their toxicity, we carried out an in vitro toxicity study of its aqueous and organic extracts on *Artemia salina* larvae and an in vivo toxicity study on Swiss Albino mice for the extracts that proved to be the most toxic in vitro. In addition to the study of the correlation between the results of the in vitro toxicity study, the antioxidant activity of this mushroom and the chemical composition of its aqueous and organic extracts, this had already been studied in our previous work [14].

## 2. Materials and Methods

### 2.1. Mushroom Sample

The mushroom *Morchella esculenta* (L.) Pers. (code: ME‐2022/04) [14] was collected from the Bab Lakhmiss forest in the province of Taza, Morocco, in the Geographical coordinates N 32°03.704′ W 004°02.834′ [15]. The mushrooms were dried in the shade with aeration. While awaiting extraction, we stored them in the dark.

### 2.2. Preparation of Extracts

In this work, we adopted two extraction methods, aqueous extraction (decoction, infusion and maceration) and organic extraction using three organic solvents of different polarity (ethanol, acetone and diethyl ether), hot (by Soxhlet) and cold (by maceration) according to the protocols used in previous work in our laboratory [16–18]. The extracts were filtered and then concentrated under vacuum using an EVA 180 Rotary Evaporator and stored at 4°C for future use.

### 2.3. In Vitro and In Vivo Toxicity Study

The toxicity study was conducted in vitro using the Brine shrimp test on the crustacean *Artemia salina*. We tested aqueous extracts (decocted, infused and macerated) and organic extracts (ethanolic extract, ethanolic macerated, acetonic extract, acetonic macerated, diethyl ether extract and diethyl ether macerated of *Morchella esculenta* (L.) Pers., whereas for acute in vivo toxicity, only the extracts that showed significant in vitro toxicity were tested, namely decocted for aqueous extracts and ethanolic extract, diethyl ether extract and diethyl ether macerated for organic extracts.

#### 2.3.1. In Vitro Toxicity on *Artemia salina* Larvae

The in vitro toxicity of aqueous and organic extracts of *Morchella esculenta* (L.) Pers. was assessed using the Brine shrimp test, as described by Meyer et al. [19]. *Artemia salina* larvae commercially available (JBL ArtemioSal) were purchased and hatched in salt water prepared by solubilising 38 g of sea salt in 1 L of distilled water [20]. The test consists of preparing a range of concentrations of each *Morchella esculenta* (L.) Pers. extract; in our study, we adopted the range 10, 50, 70, 100, 500, 700, 1000 and 3000 μg/mL for the organic extracts and 0.1, 0.2, 0.3, 0.5, 2, 4, 6, and 8 g/L for the aqueous extracts. For each concentration, we carried out three replicates; in each tube, we added 10 active larvae, which were then incubated for 24 h at 25°C. The negative control used was distilled water. We then counted the number of surviving larvae and calculated the percentage lethality using the following formula:

(1) M%=LC−LTLC×100,

*M*: mortality in %. LC: live larvae in the control after 24 h. LT: live larvae with the agent tested after 24 h.

#### 2.3.2. Acute In Vivo Toxicity

The study of the acute in vivo toxicity of *Morchella esculenta* (L.) Pers. extracts, which showed high toxicity in the Brine shrimp test, was carried out in accordance with guideline No. 423 of the Organisation for Economic Co‐operation and Development [21].

##### 2.3.2.1. Animal Material

The animals used in our study were Swiss Albino mice supplied by the animal house at the Polydisciplinary Faculty of Taza‐Sidi Mohamed Ben Abdellah University‐Fez, weighing between 25 g and 35 g. They were kept with free access to food and water under conventional standards of temperature, ventilation, light and darkness in accordance with the guideline No. 423 of the Organisation for Economic Co‐operation and Development [21].

##### 2.3.2.2. Acute Toxicity

The decocted, ethanolic extract, diethyl ether extract and diethyl ether macerated are the extracts of *Morchella esculenta* (L.) Pers. which are used after the Brine shrimp test to assess the acute toxicity of our mushroom in vivo according to the directives of guideline No. 423 of the Organisation for Economic Co‐operation and Development [21]. The test protocol has been detailed in previous work by our laboratory [22–24] and consists of administering each extract orally to three mice at a dose of 2000 mg/kg with a volume of extract of the order of 0.5 mL/20 g of body weight of the animal, while the control batch received water. It should be noted that the mice were kept fasting for 4 h before the start of the experiment with free access to water, which enabled us to estimate the value of the lethal dose 50 (LD50). The behaviour of the mice was carefully observed from the moment the extracts were administered to the animals, during the first few hours and daily for 14 days. During this phase, any signs of toxicity (e.g., circling, haematuria or mortality) were noted so that we could move on to the next stages of the experiment in accordance with OECD guideline No. 423. We also calculated the body weight gain (BWG), which represents the difference between the body weights of the mice on the last day and the first day of the experiment, using the following formula:

(2) GPC=Weightd14−Weightd0,

GPC: BWG in g. Weight (*d*_14_): weight of the mouse on the day of sacrifice. Weight (*d*_0_): weight of the mouse on the first day.

### 2.4. In Vitro Antioxidant Activity

#### 2.4.1. Hydrogen Peroxide (H_2_O_2_) Scavenging Potential

We used the method of Ruch et al. [25] to measure the ability of the extracts to scavenge hydrogen peroxide (H_2_O_2_). The protocol was detailed in previous work from our laboratory [17, 26, 27]. The formula below was used to calculate the percentage of hydrogen peroxide elimination:

(3) % inhibition=AC−AEAC×100,

AC: absorbance of the control. AE: absorbance of the sample.

#### 2.4.2. Reduction Potential of the 2,2‐Azino‐bis‐3‐ethylbenzothiazoline‐6‐sulphonic Acid (ABTS)

In this test, we adopted the method developed by Re et al. [28]. The results are expressed in milligrams of Trolox equivalent per gram of extract (mg TE/g E). Previous work carried out by our laboratory provides details of the protocol [17, 26, 27].

#### 2.4.3. Ferric‐Reducing Antioxidant Power (FRAP)

The method described by Benzie and Strain [29], in which ascorbic acid was used as a positive control, was used to evaluate the antioxidant capacity of the extracts, and the results obtained are expressed in milligrams of Trolox equivalent per gram of extract (mg TE/g E). The protocol adopted was mentioned in our previous work [17, 26, 27].

#### 2.4.4. Iron‐Reducing Power (IRP) Test

The Oyaizu method [30] was used to measure the reducing power of ferric iron to ferrous iron. The results are expressed in milligrams of ascorbic acid equivalent per gram of extract (mg AAE/g E). The protocol was detailed in previously published work by our group [17, 26, 27].

### 2.5. Statistical Analysis

Statistical analysis of the results was performed using one‐way analysis of variance (ANOVA) followed by Tukey’s test using GraphPad Prism 5 software. Results are expressed as mean ± standard error of mean (SEM), and the difference was considered statistically significant when the *p*‐value was ≤ 0.05. Analysis of the correlations between the chemical composition (total polyphenols, flavonoids and catechic tannins) of the aqueous and organic extracts of *Morchella esculenta* (L.) Pers. from Taza determined previously in our work [14], their antioxidant activity and the in vitro toxicity results was carried out using XLSTAT software.

## 3. Results

### 3.1. In Vitro Toxicity on *Artemia salina* Larvae

**Figure 1:**
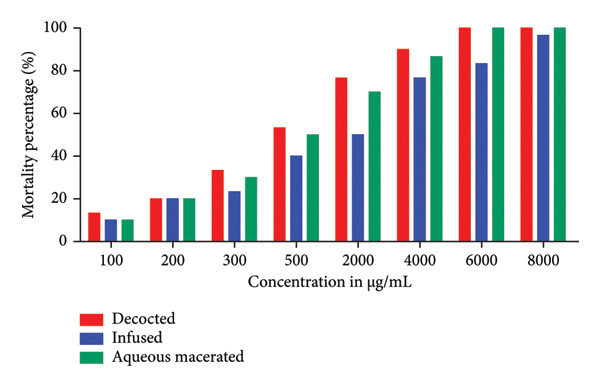
Percentage mortality of *Artemia salina* treated with aqueous extracts of *Morchella esculenta* (L.) Pers.

**Figure 2:**
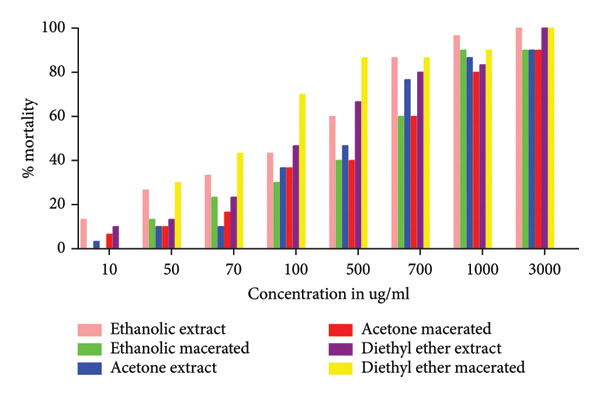
Percentage mortality of *Artemia salina* treated with organic extracts of *Morchella esculenta* (L.) Pers.

Evaluation of the toxicity of *Morchella esculenta* (L.) Pers. by the Brine shrimp lethality test using *Artemia salina* larvae showed that the percentage of lethality reached 100% with a dose of 3000 μg/mL with the ethanolic, diethyl ether and diethyl ether macerated extracts, while for the rest of the organic extracts, it reached a percentage of 90% with the same dose. For aqueous extracts, 100% is reached by the dose 6000 μg/mL for the decocted and aqueous macerated, whereas the infused reaches its maximum lethality which is of the order of 96.66% at 8000 μg/mL. Figure 1 shows the results obtained for aqueous extracts and Figure 2 shows the results for organic extracts, where we can see that the percentage mortality of our extracts increases with increasing concentration.

After calculating the percentages of mortality caused by the aqueous and organic extracts of *Morchella esculenta*, we proceeded to determine the value of the median LD50, which corresponds to the dose of a substance that causes the death of 50% of a population formed by the same animal species (in our case, the larvae of *Artemia salina*). Table 1 shows the LD50 values in μg/mL of the aqueous and organic extracts tested, with the infused extract having a high LD50 compared with the other aqueous extracts, that is, a low toxic potential with a value equal to 3443.33 ± 20.66 μg/mL, followed by the aqueous macerated with 724.03 ± 0.16 μg/mL and the decocted with 567.4 ± 7.2 μg/mL. Statistical analysis showed that there was a significant difference between the three aqueous extracts. For organic extracts, diethyl ether macerated had the lowest LD50 68.91 ± 2.76 μg/mL followed by diethyl ether extract 217.73 ± 13.68 μg/mL, ethanolic extract 297.56 ± 14.45 μg/mL, acetonic extract 326.1 ± 24.89 μg/mL, ethanolic macerated 531.6 ± 12.23 μg/mL and finally acetonic macerated 542.93 ± 17.35 μg/mL. Comparison of the LD50s of the extracts allows us to deduce that the acetone macerated extract is the least toxic compared with the other organic extracts. Statistical analysis revealed that there was a statistically nonsignificant difference between the decocted, the ethanolic macerated and the acetone macerated, a nonsignificant difference between the ethanolic extract and the acetone extract, and a statistically significant difference between the diethyl ether extract and the diethyl ether macerated. We note that aqueous and organic extracts have LD50s that vary from one extract to another and therefore toxicity that varies according to the preparation method used, hot or cold, and also according to the polarity of the solvent used for extraction.

**Table 1:** Median lethal dose (LD50) of aqueous and organic extracts of *Morchella esculenta* (L.) Pers.

Extracts	LD50 (μg/mL)
Decocted	**567.4 ± 7.2** ^**a**^
Infused	3443.33 ± 20.66^b^
Aqueous macerated	724.03 ± 0.16^c^
Ethanolic extract	**297.56 ± 14.45** ^d^
Ethanolic macerated	531.6 ± 12.23^a^
Acetone extract	326.1 ± 24.89^d^
Acetone macerated	542.93 ± 17.35^a^
Diethyl ether extract	**217.73 ± 13.68** ^e^
Diethyl ether macerated	**68.91 ± 2.76** ^f^

*Note:* The values given in Table 1 represent the mean of three replicates ± mean standard error. The difference was examined as statistically significant at *p* < 0.05. Values in bold highlight the most important results obtained.

Given that mushrooms are known for their toxicity, the above test enabled us to assess the in vitro toxicity of *Morchella esculenta* (L.) Pers. from Taza using Meyer’s classification, which considers an extract to be toxic when its LD50 is less than 1000 μg/mL, whereas an extract with an LD50 exceeding 1000 μg/mL is nontoxic [19]. And so, we can say that infused is the only extract that presents no risk of toxicity because its LD50 is of the order of 3443.33 ± 20.66 μg/mL, while diethyl ether macerated is the most toxic extract with an LD50 equal to 68.91 ± 2.76 μg/mL, while the rest of the extracts have a toxicity that is medium or moderate and we note that, generally, organic extracts can be considered more toxic than aqueous extracts.

### 3.2. Acute Toxicity

In order to minimise the number of animals used in the acute toxicity study, we tested only those extracts that showed high in vitro toxicity, that is, decocted, ethanolic extract, diethyl ether extract and diethyl ether macerated.

**Figure 3:**
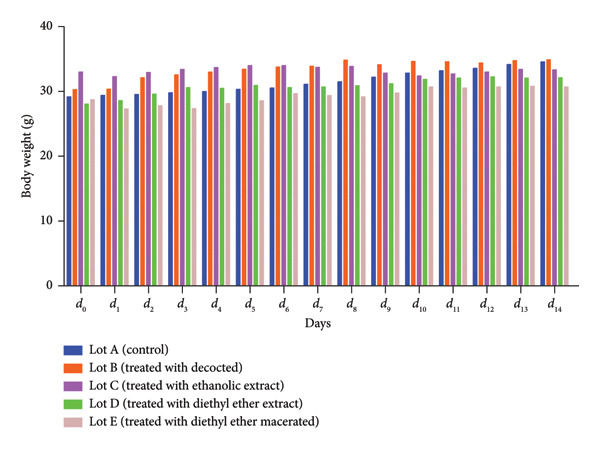
Changes in body weight of animals treated with *Morchella esculenta* (L.) Pers. decocted, ethanolic extract, diethyl ether extract and diethyl ether macerated for 14 days.

Mice in batches B, C, D and E that received the following extracts: decocted, ethanolic extract, diethyl ether extract and diethyl ether macerated, respectively, showed normal behaviour after administration of the extracts, as did mice in Batch A (control), with no mortality in mice that received *Morchella esculenta* (L.) Pers. extracts (Table 2). We also noted the absence of any clinical signs, such as respiratory distress and walking stiffly, for all the batches. Similarly, the extracts did not cause any changes in hair, skin, eye condition or the nature of stools and urine over the test period. In addition to these observations, we monitored the body weight of the mice, the results of which are illustrated in Figure 3, where we note that there was, generally, a slight increase in weight in the mice in the 4 batches that received the extracts as the control batch during the experiment period, with a statistically significant difference between the control batch and the batches that received the decocted, the ethanolic extract and the diethyl ether extract, whereas there was a statistically nonsignificant difference between the control and the batch that received the diethyl ether macerated.

**Table 2:** Mortality and clinical signs in the acute toxicity study of decocted, ethanolic extract, diethyl ether extract and diethyl ether macerated of *Morchella esculenta* (L.) Pers. on Swiss albino mice.

Swiss albino mice	Stage 1			Stage 2		
	Body weight dose (mg/kg)	Number of dead mice	Clinical signs of acute toxicity	Body weight dose (mg/kg)	Number of dead mice	Clinical signs of acute toxicity
Lot A (control)	0	0	None	0	0	None
Lot B (treated with decocted)	2000 mg/kg	0	None	2000 mg/kg	0	None
Lot C (treated with ethanolic extract)	2000 mg/kg	0	None	2000 mg/kg	0	None
Lot D (treated with diethyl ether extract)	2000 mg/kg	0	None	2000 mg/kg	0	None
Lot E (treated with diethyl ether macerated)	2000 mg/kg	0	None	2000 mg/kg	0	None

*Note:* The values given in Table 2 represent the mean of three replicates ± mean standard error. The difference was examined as statistically significant at *p* < 0.05.

The results of the BWG calculation are shown in Table 3, which shows that the weight of the mice increased after oral administration of the different *Morchella esculenta* extracts, and from Table 3, we see that decocted is the extract that gave the highest BWG (4.59 g) compared with the other extracts. Statistical analysis showed a significant difference between the control and the decocted, the ethanolic extract and the diethyl ether extract, but a statistically nonsignificant difference between the control and the batch that received the diethyl ether macerated, in addition to a statistically significant difference between Batch B (treated with decocted) and Batch C (treated with ethanolic extract) on the one hand and Batch A (control) and Batch D (treated with diethyl ether extract) on the other.

**Table 3:** Body weight gain (BWG) measurements of Swiss albino mice using extracts of *Morchella esculenta* (L.) Pers.

Extracts	BWG (g)
Lot A (control)	5.42 ± 0.79^a^
Lot B (treated with decocted)	**4.59** ± 0.37^**b**^
Lot C (treated with ethanolic extract)	0.35 ± 0.12^b^
Lot D (treated with diethyl ether extract)	4.07 ± 0.46^a^
Lot E (treated with diethyl ether macerated)	1.94 ± 0.23^c^

*Note:* The values given in Table 3 represent the mean of three replicates ± mean standard error. The difference was examined as statistically significant at *p* < 0.05. Bold values represent the most important results obtained.

### 3.3. In Vitro Antioxidant Activity

The study of the antioxidant activity of aqueous and organic extracts of *Morchella esculenta*, through four tests which are as follows: H_2_O_2_, ABTS, FRAP, and PR, yielded results that are summarised in Table 4. We find that extracts of *Morchella esculenta* (L.) Pers. have a very interesting antioxidant and radical‐scavenging potential, and it is the aqueous extracts that showed powerful activity via the hydrogen peroxide (H_2_O_2_) scavenging test mainly through decoction, the reduction of the ABTS and the FRAP test through aqueous macerated. On the other hand, it is the organic extracts that have significant IRP, mainly through diethyl ether macerated.

**Table 4:** In vitro antioxidant activity of aqueous and organic extracts of *Morchella esculenta* (L.) Pers.

Extracts		H_2_O_2_ (%)	ABTS (mg TE/g E)	FRAP (mg TE/g E)	RP (mg AAE/g E)
Extracts aqueous	Decocted	**23.69 ± 0.61** ^**a**^	166.22 ± 1.05^a^	11.16 ± 0.54^a^	0.12 ± 0.02^a^
	Infused	14.00 ± 1.44^b^	177.1 ± 0.58^b^	18.66 ± 0.50^b^	0.12 ± 0.07^a^
	Aqueous macerated	18.44 ± 0.43^ab^	**184.1 ± 0.67**​^**b**^	**137.92 ± 0.03** ^**c**^	**0.89 ± 0.08** ^**a**^

Extracts organic	Ethanolic extract	**22.71 ± 0.81** ^**a**^	46.7 ± 0.86^c^	41.74 ± 0.78^d^	1.04 ± 0.05^a^
	Ethanolic macerated	21.79 ± 0.5^a^	80.01 ± 1.66^c^	25.90 ± 0.13^e^	10.64 ± 0.22^b^
	Acetone extract	15.14 ± 1.00^b^	74.11 ± 1.63^cd^	20.33 ± 0.83^df^	16.17 ± 0.14^c^
	Acetone macerated	16.06 ± 0.32^b^	**101.88 ± 1.25** ^**e**^	36.037 ± 1.13^g^	16.20 ± 0.24^c^
	Diethyl ether extract	18.78 ± 0.36a^b^	**102.5 ± 1.65** ^**e**^	**52.01 ± 0.73** ^**h**^	23.53 ± 0.1^d^
	Diethyl ether macerated	12.36 ± 0.24^b^	89.11 ± 0.94^ef^	35.11 ± 1.56^i^	**43.76 ± 0.51** ^**e**^

Reference standards	Ascorbic acid	14,35 ± 0,002^b^	—	—	—
	Trolox	—	—	—	—

*Note:* The values mentioned in Table 4 are the average of three repetitions ± the average of the standard error. In the same column (same test), extracts with values having the same letter do not differ statistically (*p* < 0.05). mg AAE/g E: milligrams of ascorbic acid equivalent per gram of extract. mg TE/g E: milligrams of Trolox equivalent per gram of extract. Bold values represent the most important results obtained.

#### 3.3.1. Hydrogen Peroxide (H_2_O_2_) Scavenging Potential

For the trapping of hydrogen peroxide (H_2_O_2_), we notice that the decoction is the most active extract with a percentage of 23.69 ± 0.61%, followed by the aqueous macerated at 18.44 ± 0.43%, and the infusion comes last at 14.00 ± 1.44%. The statistical analysis showed that the difference between the decoction and the infusion is statistically significant, whereas it is not significant between the infused and the aqueous macerated. However, the ethanolic extract has the highest trapping potential at 22.71 ± 0.81% compared to other organic extracts, followed by the ethanolic macerated (21.79 ± 0.5%), the diethyl ether extract (18.78 ± 0.36%), the acetonic macerated (16.06 ± 0.32%), the acetonic extract (15.14 ± 1.00%) and finally the diethyl ether macerated (8.36 ± 0.24%), with a statistically nonsignificant difference between the ethanolic extract and the ethanolic macerated on one side and the acetonic extract, the acetonic macerated, the diethyl ether extract and the diethyl ether macerated on the other side. It should be noted that the standard antioxidant (ascorbic acid) has a statistically nonsignificant difference with the infusion, the aqueous macerated, the acetonic extract, the acetonic macerated, the diethyl ether extract and the diethyl ether macerated, whereas it has a statistically significant difference with the decoction, the ethanolic extract and the ethanolic macerated. It should be noted here that the activity of the decocted aqueous extract exceeds that of the organic extracts.

#### 3.3.2. Reduction Potential of the ABTS

Through this test, we found that the aqueous macerated is the extract with the highest reduction activity (184.1 ± 0.67 mg E AA/gE), followed by the infusion (177.1 ± 0.58 mg E AA/gE) and then the decoction (166.22 ± 1.05 mg E AA/gE), with a statistically significant difference between the decoction and the infusion, and no significant difference between the latter and the aqueous macerated. For the organic extracts, it is the diethyl ether extract (102.5 ± 1.65 mg E AA/gE) and the acetonic macerated (101.88 ± 1.25 mg E AA/gE) that are the most active. After that, we find the other extracts: diethyl ether macerated > ethanolic macerated > acetonic extract > ethanolic extract, which have the following values of 89.11 ± 0.94, 80.01 ± 1.66, 74.11 ± 1.63 and 46.7 ± 0.86 mg E AA/gE, respectively. The statistical analysis showed that there is a statistically nonsignificant difference between the ethanolic extract, the ethanolic macerated and the acetonic extract, whereas they have a statistically significant difference with the rest of the extracts.

#### 3.3.3. FRAP

The FRAP of *Morchella esculenta* extracts is particularly interesting, especially that of the aqueous macerated, which has a value of around 137.92 ± 0.03 mg TE/gE, followed by the infusion and decoction, which are 18.66 ± 0.50 and 11.16 ± 0.54 mg TE/gE, respectively, with a statistically significant difference between the three aqueous extracts. Regarding the organic extracts, we have obtained the following ranking: diethyl ether extract > ethanolic extract > acetonic macerated > diethyl ether macerated > ethanolic macerated > acetonic extract which have the following values: 52.01 ± 0.73, 41.74 ± 0.78, 36.037 ± 1.13, 35.11 ± 1.56, 25.90 ± 0.13 and 20.33 ± 0.83 mg ET/g, respectively, and all these extracts show a statistically significant difference between them except for the ethanolic and acetonic extracts.

#### 3.3.4. IRP Test

The IRP of the aqueous extracts of *Morchella esculenta* is low, where we observe that the aqueous macerated is the most active at 0.89 ± 0.08 mg E AA/gE, followed in second place by the decoction and the infusion at 0.12 ± 0.02 and 0.12 ± 0.07 mg E AA/gE, respectively, and the difference between the three extracts is statistically nonsignificant. On the other hand, we observe a considerable increase in activity among the organic extracts, with the diethyl ether macerated in first position, followed by the diethyl ether extract, the acetonic macerated, the acetonic extract, the ethanolic macerated and the ethanolic extract, with values of 43.76 ± 0.51, 23.53 ± 0.1, 16.20 ± 0.24, 16.17 ± 0.14, 10.64 ± 0.22 and 1.04 ± 0.05 mg E AA/gE, respectively, with a statistically significant difference between the ethanolic extract, the ethanolic macerated, the diethyl ether extract and the diethyl ether macerated, while the difference between the acetonic extract and the acetonic macerated is statistically nonsignificant.

### 3.4. Principal Component Analysis (PCA)

PCA using Pearson correlation (*r*) allowed us to study the correlation between individuals represented by *Morchella esculenta* extracts and the variables which are the contents of chemical compounds (total polyphenols, flavonoids and catechin tannins) in the aqueous and organic extracts of *Morchella esculenta* (L.) Pers. from our previous study [14], the antioxidant capacity measured by the four tests H_2_O_2_, ABTS, FRAP and PR in the present study, and the results of in vitro toxicity.

#### 3.4.1. Correlation Matrix

The degree of relationship between each pair of variables used in this study is visualised by the correlation matrix presented in Table 5, where we observe a significant correlation between polyphenols and the FRAP test (*r* = 0.8369) and a strong correlation between flavonoids and the PR test (*r* = 0.8484). Flavonoids were also strongly correlated with catechin tannins (r = 0.8944), and catechin tannins showed a strong correlation with the PR test. In contrast, polyphenols showed a weak correlation with the ABTS test (r = 0.2705), and a moderate negative correlation was observed between the H_2_O_2_ test and flavonoids (r = −0.5217), and between H_2_O_2_ and catechin tannins (r = −0.3679). Finally, a moderate positive correlation was found between the FRAP and ABTS tests (*r* = 0.3430). We found that there is a strong correlation between in vitro toxicity with catechin tannin content and reducing power, which are *r* = 0.8079 and *r* = 0.7869, respectively.

**Table 5:** Correlation matrix between phytochemical data (total polyphenol content, flavonoids and catechin tannins) and antioxidant activity via the four tests (H_2_O_2_, ABTS, FRAP and RP) of *Morchella esculenta* (L.) Pers.

Variables	Polyphenols	Flavonoids	Catechic tannins	H_2_O_2_	ABTS	FRAP	PR	In vitro toxicity
Polyphenols	**1**							
Flavonoids	0.0249	**1**						
Catechic tannins	0.2041	**0.8944**	**1**					
H_2_O_2_	0.0813	−0.5217	−0.3679	**1**				
ABTS	0.2705	−0.3193	−0.5707	−0.0630	**1**			
FRAP	**0.8369**	−0.0642	0.1020	0.0147	**0.3430**	**1**		
PR	0.0358	**0.8484**	**0.8729**	−0.5805	−0.4312	−0.1387	**1**	
In vitro toxicity	0.1944	0.5994	**0.8079**	−0.0792	−0.6266	0.0002	**0.7869**	**1**

*Note:* Bold values represent the most important results obtained.

#### 3.4.2. Graphical Representation of PCA

**Figure 4:**
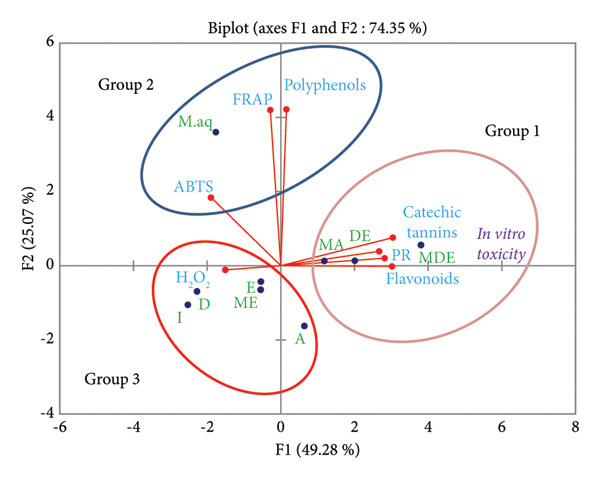
Projection of individuals on the factorial plane (F1 X F2). **D**: decoction; **I**: infusion; **M.aq**: aqueous macerated; **E**: ethanol extract; **ME**: ethanol macerated; **A**: acetone extract; **MA**: acetone macerated; **DE**: diethyl ether extract; **MDE**: diethyl ether macerated.

The analysis conducted by PCA on the two axes F1 and F2 yielded a cumulative percentage of approximately 75.79% of the information from the initial database, with F1 providing 47.25% of the total information and F2 providing 28.53% (Figure 4). The visualisation of the data on the two axes F1 and F2 allowed us to identify 3 groups:

• **Group 1**: formed by acetonic macerated, diethyl ether extract, diethyl ether macerated, flavonoids, catechin tannins, PR test and in vitro toxicity. This assembly confirms the experimental results because the three extracts in this group have the highest levels of flavonoids and catechin tannins and the most significant reducing potential, obtained through the PR test, compared to the other tested extracts. Moreover, the diethyl ether extract and the diethyl ether macerated have the lowest DL 50 values, indicating high in vitro toxicity.
• **Group 2**: includes the aqueous macerated, polyphenols, the FRAP test and the ABTS test, which may be due to the fact that the aqueous macerated is the extract richest in polyphenols, and it exhibited very high antiradical activity via the ABTS test and intense reducing power by the FRAP test.
• **Group 3**: comprises decocted, infused, ethanolic extract, ethanolic macerate and acetone extract and the H_2_O_2_ test. The decocted is the most active (23.69 ± 0.6%) in the H_2_O_2_ test, and this activity decreases in the other extracts: infused (14.00 ± 1.44%), ethanolic extract (22.71 ± 0.81%), ethanolic macerated (21.79 ± 0.5%) and acetone extract (15.14 ± 1.00%).

## 4. Discussion

### 4.1. In Vitro Toxicity on *Artemia salina* Larvae

The (BS) test can be used for several purposes, such as studying the general toxicity, assessing the cytotoxic potential and pesticidal activity of substances [31]. Since mushrooms are known for their toxicity, this test enabled us to assess the in vitro toxicity of *Morchella esculenta* (L.) Pers. from Taza using Meyer’s classification, which considers that an extract is toxic when its LD50 is less than 1000 μg/mL, whereas an extract with an LD50 that exceeds 1000 μg/mL is nontoxic [19]. The results of this study showed that the infused extract presents no risk of toxicity, with an LD50 of 3443.33 ± 20.66 μg/mL, while the diethyl ether macerate is the most toxic extract, with an LD50 of 68.91 ± 2.76 μg/mL, and the latter could be considered harmless to living cells [32]. On the other hand, the rest of the extracts were moderately to moderately toxic. We found that the toxicity of the organic extracts exceeded that of the aqueous extracts, which may be due to their alkaloid content [14], and this is confirmed by previous studies [33–37].

Comparing the results of the present study with those obtained by Qwarse et al. [38] who used the Brine shrimp test to study the toxicity of an edible mushroom which is named *Afrocantharellus platyphyllus* (Heinem.) Tibuhwa, their ethanolic extract showed high toxicity (61.94 μg/mL) compared to the ethanolic extract of *Morchella esculenta* (297.56 ± 14.45 μg/mL) in our study. While the study conducted by Masalu [31] on the methyl acetate extract of some mushroom which are basidiomycetes, zygomycetes and ascomycetes, this study showed that the basidiomycete (*Lactarius volemoides*) is a highly toxic mushroom with a lethal concentration that kills 50% of *Artemia salina* larvae equal to 0, 28 μg/mL, while the ascomycete *Pichia guilliermondii* is less toxic (LD50 = 40 μg/mL) and both are toxic compared with the *Morchella esculenta* extracts tested.

### 4.2. Acute Toxicity

After administration of extracts: decoction, ethanol extract, diethyl ether extract and diethyl ether macerate of *Morchella esculenta* (L.) Pers. to mice that survived after 14 days of follow‐up, the LD50 is estimated at 5000 mg/kg body weight according to the method described in OECD Guideline No. 423 [27]. We can therefore classify our extracts as category five or unclassified. The results of this study are in accordance with those obtained by Das in 2025 on *Morchella esculenta* (L.) Pers. [39]. Our results were also compared with acute toxicity data for certain plant materials collected in the Taza region of Morocco and studied in our laboratory (SNAMOPEQ), such as *Haloxylon scoparium* Pomel, whose extracts have relatively low acute toxicity and an estimated LD50 of 2000 mg/kg [22], while the species *Ajuga iva* has an estimated LD50 greater than 2000 mg/kg [23].

### 4.3. In Vitro Antioxidant Activity

#### 4.3.1. Hydrogen Peroxide (H_2_O_2_) Scavenging Potential

Based on the results of this study, it can be said that the most effective extraction method for obtaining an interesting potential for trapping hydrogen peroxide (H_2_O_2_) is that involving heat treatment (by decoction or Soxhlet) and the use of water or a polar organic solvent (ethanol).

The results of the study conducted on *Morchella esculenta* from the north‐western region of the Himalayas, in Shimla, India [40], showed that the potential for trapping the H_2_O_2_ radical of the methanolic extract was equal to 84.12 ± 0.2810%, which surpasses the potential of the most active organic extract of *Morchella esculenta* from Taza, Morocco, which is the ethanolic extract (22.71 ± 0.81%). The study conducted by Anand and his colleagues, who performed the H_2_O_2_ scavenging test to test the antiradical potential of the ethanolic extract, chloroform extract and methanolic extract of *Morchella esculenta*, showed that the ethanolic extract was the most active [41]. This difference in the results of these studies may be due to the nature and chemical properties of the solvents used for extraction, as well as differences in environmental conditions at the harvesting site [42]. The potential of hydrogen peroxide (H_2_O_2_) trapping was explained by the study conducted by Zhang and his collaborators [43] on *Morchella esculenta*, which showed that *Morchella* protein hydrolysate (MPH) and its glycosylated derivative (G‐MPH) have the ability to mitigate the effects of H_2_O_2_ in cells and demonstrated protective effects by inhibiting the production of reactive oxygen species (ROS) and malondialdehydes (MDA).

Comparing our results with the work of our laboratory conducted on plants from the Taza region, Morocco, we find that *Morchella esculenta* has a lower hydrogen peroxide (H_2_O_2_) trapping capacity than *Chamaerops humilis* L. var. argentea Andre Leaves [42]. This study revealed significant H_2_O_2_ trapping activity with the decoction and the ethanolic extract, which are 45.77 ± 0.15 and 36.80 ± 0.33 4%, respectively, which aligns with our results. Boulfia and his collaborators in 2021 studied the antioxidant activity of the bulb of *Leopoldia comosa* (L.) from the Taza region, Morocco [25], and found that the decoction is the most active aqueous extract at 62.12 ± 0.2%, which is similar to our results, whereas the active organic extract is the one obtained with the nonpolar solvent diethyl ether at 62.67 ± 0.06%, which differs from our results. We also found that our results are consistent with those obtained by a study conducted on *Haloxylon scoparium* [27], where the decoction and the methanolic extract (extract prepared with a polar solvent) have the highest activity, with percentages of 16.21 ± 0.39% and 20.91 ± 0.27%, respectively, which means that these two extraction methods are the most effective for extracting antioxidant substances from this plant. While the three studies conducted on *Atractylis gummifera* [44], *Ajuga iva* subsp. Pseudoiva [17] and *Anabasis aretioïdes* Coss. & Moq. [45] found that the aqueous macerated and the methanolic macerated are the most active extracts, and this can be explained by the chemical nature of the bioactive substances present in the three plants.

#### 4.3.2. Reduction Potential of the ABTS

Based on the results of this test, we detected that aqueous extraction could be very effective for extracting antioxidant factors from *Morchella esculenta*, especially without heat treatment, that is, by simple maceration.

Our results are consistent with the results of the ABTS test conducted by Dimitrijevic and his collaborators [46] on the ethanolic extract of *Morchella esculenta* from the city of Nis in the Republic of Serbia, which showed that this mushroom has an interesting potential for trapping ABTS with an effective concentration of 50% inhibition (EC50) around 5 mg/mL. The study conducted by Badshah and his collaborators [47] proves that the polysaccharides of *Morchella esculenta* have antioxidant activity through hydrogen atom donor antioxidants and chain‐breaking antioxidants.

For our study, it is the aqueous macerated followed by the acetonic macerated and the diethyl ether extract that revealed an interesting antioxidant power. However, based on the comparison of our results with those of the studies conducted within our laboratory, we found that according to the study carried out by Lachkar and his collaborators [42] on an edible plant from the Taza region, which is *Chamaerops humilis* L. var. argentea Andre Leaves, this plant has intense activity through organic extracts, specifically the ethanolic macerated (108.28 ± 1.29 mg TE/g E), which exceeds that of aqueous extracts. This may be due to the nature of the active principles present in the tested material. On the other hand, the ABTS test conducted on extracts of *Atractylis gummifera* from Taza [44] revealed that the methanolic macerated has the highest antioxidant capacity, followed by the aqueous macerated, meaning that cold extraction with water and the most polar solvent is the appropriate method to benefit from substances with antioxidant potential. For *Leopoldia comosa* (L.) from Taza, the most effective extraction method was the hot aqueous extraction (decoction) and hot extraction with the nonpolar solvent diethyl ether [26]. On the other hand, the aqueous extracts of *Anabasis aretioides* are less active compared to the organic extracts obtained with polar organic solvents [45]. These studies confirm that the extraction method adopted to extract antioxidant substances could be correlated with the chemical properties of these substances [47].

#### 4.3.3. FRAP

Based on the data obtained from this test, we found that antioxidant power varies depending on the nature of the solvent, which is consistent with the results obtained by Hira and his colleagues [48]. We also found that cold aqueous extraction is the most effective method for extracting the iron‐reducing antioxidants present in *Morchella esculenta* (L.) Pers.

By comparing the results of our work obtained for the ethanolic extract (41.74 ± 0.78 mg TE/g E) with those of Dimitrijevic and his collaborators [46], who conducted the FRAP test on the ethanolic extract of *Morchella esculenta* from the city of Nis, Republic of Serbia, with a result of 23.347 ± 0.006 μmol Fe/1 mg dw, we observe a difference in the potency of the two mushrooms, despite the study protocol being the same, which can be explained by the difference in the period and area of mushroom harvesting in the two studies.

By comparing the FRAP of Morchella esculenta from Taza with that of plants from the same region, which are studied in our laboratory using the same protocol and under the same conditions, which are Leopoldia comosa (L.) [20], we observe a similar trend, in which the aqueous macerate and the diethyl ether extract exhibit the most active extracts. *Atractylis gummifera* [44] showed results consistent with ours, where water maceration was the most effective extraction method (aqueous), while their active organic extract was the methanolic macerated, that is, cold extraction with a polar organic solvent. On the other hand, for the mushroom *M. esculenta*, we found that hot organic extraction with a nonpolar solvent provided the most interesting potency compared to polar solvents. We found that the most active extracts of Ajuga iva Subsp. Pseudoiva [17] are the decocted (6.86 µg TE/mg E) and the methanolic extracts (22.70 µg TE/mg E). Thus, we can say that the potency of *Ajuga iva* Subsp. Pseudoiva extracts is very low compared to the antioxidant power of the mushroom in the present study. The extracts obtained from decoction and maceration with a polar solvent (ethanol) of *Chamaerops humilis* [42] have demonstrated high activity, which differs from what is obtained with our extracts.

#### 4.3.4. IRP Test

The present work has shown that the cold extraction method (maceration) and also the low polarity of the organic solvent used for extraction (diethyl ether) make it possible to extract the agent(s) with IRP (transformation of ferric iron into ferrous iron).

The study conducted on the ethanolic extract of *Morchella esculenta* from Serbia [46] showed a reducing power of approximately 0.449 ± 0.010 mg EAA/1 mg dw, equivalent to 449 mg AAE/g dw, which exceeds the power of *Morchella esculenta* from Taza obtained by the same extract (1.04 ± 0.05 mg EAA/gE). Knowing that both studies used the same Oyaizu method [31], but the PR potential is different, this difference may be due to the difference in the mushroom harvesting period, as for our study, the harvest was carried out in April, whereas the harvest of *Morchella esculenta* in Serbia was conducted during the months of July and August, and to the climatic and geographical differences that exist between Morocco and Serbia, which influence the antiradical and antioxidant properties.

The study conducted in our laboratory by Boulfia et al. on the reducing power of extracts from *Leopoldia comosa* (L.) Parl. from the Taza region [26], yielded results that are consistent with ours, where the aqueous macerate was the most active aqueous extract, but this activity was surpassed by that of the extraction with the nonpolar solvent (diethyl ether). It should be noted that *Leopoldia comosa* showed a very interesting reducing power compared to *Morchella esculenta* from Taza.

*Atractylis gummifera* from Taza [44] also showed high activity with the aqueous macerated, but for organic extraction, the extract prepared by maceration with a polar solvent (methanolic macerated) was the most active. On the other hand, *Chamaerops humilis* [42] revealed antioxidant activity through its two extracts: the infusion and the ethanolic macerated, but this activity is weak compared to the activity of *Morchella esculenta*. While the potency of *Anabasis aretioïdes* [45] has been elevated at the level of organic extracts, which are the methanolic extract, the methanolic macerated, and the ethyl acetate extract, *Haloxylon scoparium* [27] has shown a powerful effect in the decoction as well as in the extract obtained from hot polar solvent, which is the methanolic extract.

## 5. PCA

The PCA revealed a moderate positive correlation between the FRAP and ABTS tests, while the PR and H_2_O_2_ tests were negatively correlated. We also observe the presence of a positive correlation between the FRAP and ABTS tests and polyphenols, which is in agreement with the study conducted by Jafri and his collaborators and the work of Ribeiro and his colleagues [49, 50]. These three variables are correlated with the aqueous macerated, which could be due to the richness of the aqueous macerated in polyphenols [14] and the presence of active principles that have antiradical activity toward the ABTS and have the ability to reduce Fe (III) based on electron transfer [29].

The PCA showed that there is a positive correlation between the following extracts: acetonic macerated, diethyl ether extract and diethyl ether macerated, the PR test and flavonoids, which can be explained as follows: these extracts contain a significant amount of flavonoids according to our previous study [14] and these extracts also have antioxidant properties, and this correlation may be due to the antioxidant impact of flavonoids [51, 52].

The study conducted on the mushroom *Schizophyllum commune* [53] confirms the presence of a strong positive correlation between antioxidant activity and the content of phenolic compounds, and this activity may be due to the ability of phenolic compounds to scavenge hydroxyl groups. For Gursoy and his collaborators [54] who studied the antioxidant activity and chemical composition of seven species of morels, the methanolic extract of *Morchella esculenta* var. umbrina showed significant antioxidant activity compared to the other species with the three ABTS tests (76.38 ± 1.61%) at a concentration of 40 μg/mL, *β*‐carotene/linoleic acid (96.89 ± 0.34%) and DPPH (62.57 ± 1.83%) at a concentration of 4.5 mg/mL. Additionally, this study considers that flavonoids, phenolic acids and tannins are contributors to the antioxidant effect. Among the phenolic compounds characterised by high antioxidant activity are malvin, callistephine, silychristin and 3,4‐dihydroxy‐5‐methoxybenzoic acid [55].

Recent studies conducted in our laboratory on plants from the Taza region, Morocco, have demonstrated a correlation between chemical composition and antioxidant activity; for example, the study conducted on *Leopoldia comosa* (L.) Parl. by Boulfia and his collaborators [26], who found a positive correlation between the antioxidant activity evaluation tests ABTS, FRAP and PR and the content of polyphenols, flavonoids and catechin tannins, in addition to the study conducted by Lachkar and his collaborators on *Chamaerops humilis* L. var. argentea Andre Leaves [42], whose results show a positive correlation between the ABTS, FRAP and PR tests and the content of polyphenols, flavonoids and catechin tannins. This was also confirmed by the study by Senhaji and his collaborators [44], which showed that the polyphenol content of *Anabasis aretioides* is correlated with the ABTS, FRAP and PR tests.

This is affirmed by the study conducted on *Haloxylon scoparium* [27]. The study conducted on *Ajuga iva* [17] revealed a positive correlation between the content of polyphenols, flavonoids and catechin tannins and the ABTS test, as well as a correlation between the FRAP, H_2_O_2_ and PR tests. We can thus say that the correlations revealed between the chemical composition and the antioxidant potential evaluated by the ABTS, FRAP, H_2_O_2_ and PR tests could be explained by the presence of one or more chemical compounds that are part of polyphenols, flavonoids and catechin tannins, which have the ability to both stabilise the ABTS cationic radical by proton trapping by the antioxidant agent and reduce iron (conversion of ferric iron to ferrous iron or iron salt).

The present study also revealed the presence of a positive correlation between phenolic compounds, specifically flavonoids and tannins, and the extracts that showed high toxicity in vitro as evaluated by the Brine shrimp test, which is in agreement with the results of the study conducted by Braguini and his collaborators [56], which revealed that the extract that is highly toxic to *Artemia salina* is the one that has significant levels of phenolic compounds. Similarly, the study conducted on the stem of *Randia dumetorum* illustrates that the extracts of the stem, which exhibited high antioxidant potential, are rich in flavonoids and polyphenols and also have a significant toxic effect as evaluated by the lethality test on *Artemia salina* larvae [57].

## 6. Conclusion

The present work, conducted for the first time, aimed to evaluate the in vitro and in vivo toxicity and antioxidant potential of aqueous extracts (decocted, infused and macerated) and organic extracts (ethanolic extract, ethanolic macerated, acetonic extract, acetonic macerated, diethyl ether extract and diethyl ether macerated) of the edible mushroom *Morchella esculenta* (L.) Pers. wild from the Taza province, Morocco. The results revealed that the infusion poses no risk of in vitro toxicity, while the diethyl ether macerated is the most toxic extract. The rest of the extracts have moderate or average toxicity. The in vivo evaluation of the acute toxicity of *Morchella esculenta* showed that the decoction, the ethanolic extract, the diethyl ether extract and the diethyl ether macerated did not cause either the death of the animals or the appearance of signs of intoxication in them, nor a weight loss. On the contrary, we observed a slight increase in body weight, especially in the group treated by gavage with the decoction. The four tests ABTS, H_2_O_2_, FRAP and PR conducted to evaluate antioxidant activity showed that the aqueous macerated possesses both antiradical activity through ABTS and reducing power via the FRAP test. In contrast, the decoction was the most active on hydrogen peroxide H_2_O_2_, while the diethyl ether macerated was the most powerful in the PR test.

PCA indicates the presence of a positive correlation among the PR test, in vitro toxicity, flavonoids, cachectic tannins, acetonic macerated, diethyl ether extract and diethyl ether macerated. It also shows a correlation among the ABTS, FRAP tests, polyphenols and aqueous macerated. In addition, a correlation is observed between the H_2_O_2_ test and the following extracts: decocted, infused, ethanolic extract, ethanolic macerated and acetonic extract.

Thus, we can conclude that the consumption of this mushroom is safe, especially after thermal treatment, and it can also be a source of antioxidants beneficial for health. The results obtained are encouraging and prompt us to conduct studies on other biological activities of *Morchella esculenta* and even to pursue in vivo studies for the extracts that revealed interesting in vitro activity.

## Conflicts of Interest

The authors declare no conflicts of interest.

## Funding

This work did not receive funding from any public, commercial or nonprofit sectors.

## Data Availability

All data generated and analysed in this study are included in this article in the form of tables and have not been deposited in public archives. The data can be requested from the corresponding author.

## References

[1] Leverve X. , Stress Oxydant Et Antioxydants?, Cahiers de Nutrition et de Diététique. (2009) 44, no. 5, 219–224, 10.1016/j.cnd.2009.09.001, 2-s2.0-73249141689.

[2] Costa T. J. , Barros P. R. , Arce C. et al., The Homeostatic Role of Hydrogen Peroxide, Superoxide Anion and Nitric Oxide in the Vasculature, Free Radical Biology and Medicine. (2021) 162, 615–635, 10.1016/j.freeradbiomed.2020.11.021. 33248264

[3] Favier A. , Stress Oxydant Et Pathologies Humaines, Annales Pharmaceutiques Françaises. (2006) 64, no. 6, 390–396, 10.1016/S0003-4509(06)75334-2. 17119468

[4] Koechlin-Ramonatxo C. , Oxygène, Stress Oxydant Et Supplémentations Antioxydantes Ou Un Aspect Différent De La Nutrition Dans Les Maladies Respiratoires, Nutrition Clinique et Metabolisme. (2006) 20, no. 4, 165–177, 10.1016/j.nupar.2006.10.178, 2-s2.0-33845382695.

[5] Valko M. , Rhodes C. J. B. , Moncol J. , Izakovic M. M. , and Mazur M. , Free Radicals, Metals and Antioxidants in Oxidative Stress-Induced Cancer, Chemico-Biological Interactions. (2006) 160, no. 1, 1–40, 10.1016/j.cbi.2005.12.009, 2-s2.0-32444433202. 16430879

[6] Govorushko S. , Rezaee R. , Dumanov J. , and Tsatsakis A. , Poi-Soning Associated with the Use of Mushrooms: A Review of the Global Pattern and Main Characteristics, Food and Chemical Toxicology. (2019) 128, 267–279, 10.1016/j.fct.2019.04.016, 2-s2.0-85064414819. 30995515

[7] Meenu M. and Xu B. , Application of Vibrational Spectroscopy for Classification, Authentication and Quality Analysis of Mushroom: A Concise Review, Food Chemistry. (2019) 289, 545–557, 10.1016/j.foodchem.2019.03.091, 2-s2.0-85063286608. 30955647

[8] Niego A. G. , Rapior S. , Thongklang N. et al., Macrofungi as a Nutraceutical Source: Promising Bioactive Compounds and Market Value, Journal of Fungi. (2021) 7, no. 5, 1–32, 10.3390/jof7050397. PMC8161071 34069721

[9] Corpuz J. C. B. , Cruz J. G. D. , Magallanes R. , and Laforteza J. V. R. , A Review on Benefits, Cultivation and Biodiversity of Macrofungi, International Journal of Scientific and Research Publication. (2023) 4, no. 11, 1586–1592, 10.55248/gengpi.4.1123.113103.

[10] Hussain S. , Sher H. , Ullah Z. et al., Traditional Uses of Wild Edible Mushrooms Among the Local Communities of Swat, Pakistan, Foods. (2023) 12, no. 8, 1–18, 10.3390/foods12081705. PMC10137476 37107503

[11] Mourid I. , Apports Des Champignons Dans Le Régime Alimentaire, Université Mohammed V de Rabat faculté de médecine et de pharmacie Rabat, Maroc. (2023) .

[12] Berthélémy S. , Intoxication Après Consommation De Champignons, Actualités Pharmaceutiques. (2014) 53, no. 538, 39–43, 10.1016/j.actpha.2014.06.009, 2-s2.0-84957311070.

[13] Flesch F. and Saviuc P. , Intoxications Par Les Champignons: Principaux Syndromes Et Traitement, EMC: Médecine. (2004) 1, no. 1, 70–79, 10.1016/j.emcmed.2003.10.002, 2-s2.0-33645727471.

[14] Mahtal A. , Lamchouri F. , and Toufik H. , Mineral Composition, Phenolic Content and Antibacterial Activity of Aqueous and Organic Extracts of Wild *Morchella esculenta* (L.) Pers. from the Province of Taza, Morocco, Biochemistry Research International. 2024, no. 1, 1–15, 10.1155/bri/6769243. PMC12714112 41425864

[15] Mahtal A. , Lamchouri F. , and Toufik H. , *Morchella esculenta* (L.) Pers. Wild of the Province of Taza, Morocco: Ethnomedicinal and Socio-Economic Survey and Perspectives, Tropical Journal of Natural Product Research. (2024) 8, no. 11, 9019–9026, 10.26538/tjnpr/v8i11.9.

[16] Bouabid K. , Lamchouri F. , Toufik H. , Sayah K. , Cherrah Y. , and Faouzi M. E. A. , Phytochemical Screening and *in Vitro* Evaluation of Alpha Amylase, Alpha Glucosidase and Beta Galactosidase Inhibition by Aqueous and Organic *Atractylis gummifera* L. Extracts, Plant Science Today. (2018) 5, no. 3, 103–112, 10.14719/pst.2018.5.3.393, 2-s2.0-85063735658.

[17] Senhaji S. , Lamchouri F. , Bouabid K. et al., Phenolic Contents and Antioxidant Properties of Aqueous and Organic Extracts of a Moroccan *Ajuga Iva* Subsp, Journal of Herbs, Spices, & Medicinal Plants. (2020) 26, no. 3, 248–266, 10.1080/10496475.2019.1709249.

[18] Boulfia M. , Lamchouri F. , and Toufik H. , Chemical Analysis, Phenolic Content, and Antioxidant Activities of Aqueous and Organic Moroccan *Juglans regia* L. Bark Extracts, Current Bioactive Compounds. (2020) 16, no. 9, 1328–1339, 10.2174/1573407216666200203103354.

[19] Meyer B. N. , Ferrigni N. R. , Putnam J. E. , Jacobsen L. B. , Nichols D. E. J. , and McLaughlin J. L. , Brine Shrimp: A Convenient General Bioassay for Active Plant Constituents, Planta Medica. (1982) 45, no. 05, 31–34, 10.1055/s-2007-971236. 7100305

[20] Ramachandr S. , Vamsikrish M. , Gowthami K. V. , Heera B. , and Dhanaraju M. D. , Assessment of Cytotoxic Activity of *Agave Cantula* Using Brine Shrimp (*Artemia salina*) Lethality Bioassay, Asian Journal of Scientific Research. (2011) 4, 90–94, 10.3923/ajsr.2011.90.94, 2-s2.0-78649770971.

[21] Organisation for Economic Co-operation and Development (OECD) , Test no 423: Acute Oral Toxicity-Acute Toxic Class Method, OECD Guidelines for the Testing of Chemicals, Section 4. (2001) Éditions OCDE.

[22] Lachkar N. , Lamchouri F. , Bouabid K. et al., *In Vitro* Study of the Antimitotic Power and *in Vivo* Acute Toxicity of Aqueous and Organic Extracts of the Aerial Part of *Haloxylon scoparium* Pomel. and Evaluation of the Correlation Between the Chemical Profile and Their Biological Activities, Plant Science Today. (2022) 10, no. 1, 242–251, 10.14719/pst.2001.

[23] Senhaji S. , Lamchouri F. , Boulfia M. et al., Cell Growth Inhibition, Toxicity Assessment, and Correlation Between Chemical Composition of Aqueous and Organic Extracts of *Ajuga Iva* Subsp. Pseudoiva (DC.) Bric. and Their Biological Activities, Biointerface Research in Applied Chemistry. (2022) 13, no. 1, 1–13, 10.33263/BRIAC131.058.

[24] Lachkar N. , Lamchouri F. , and Toufik H. , *In Vitro* Antimitotic and Hypoglycemic Effect Study and Acute Toxicity Assessment of the Aqueous and Organic Extracts of *Chamaerops humilis* L. Var. Argentea Andre, BioMed Research International. (2022) 2022, no. 1. 10.1155/2022/4303506 PMC9586795 36277886

[25] Ruch R. J. , Cheng S. J. , and Klaunig J. E. , Prevention of Cytotoxicity and Inhibition of Intercellular Communication by Antioxidant Catechins Isolated from Chinese Green Tea, Carcinogenesis. (1989) 10, no. 6, 1003–1008, 10.1093/carcin/10.6.1003, 2-s2.0-0024368805. 2470525

[26] Boulfia M. , Lamchouri F. , Senhaji S. , Lachkar N. , Bouabid K. , and Toufik H. , Mineral Content, Chemical Analysis, *in Vitro* Antidiabetic and Antioxidant Activities, and Antibacterial Power of Aqueous and Organic Extracts of Moroccan *Leopoldia comosa* (L.) Parl. Bulbs, Evidence-based Complementary and Alternative Medicine. (2021) 2021, 1–17, 10.1155/2021/9932291. PMC8324349 34335845

[27] Lachkar N. , Lamchouri F. , Bouabid K. et al., Mineral Composition, Phenolic Content, and *in Vitro* Antidiabetic and Antioxidant Properties of Aqueous and Organic Extracts of *Haloxylon scoparium* Aerial Parts, Evidence-based Complementary and Alternative Medicine. (2021) 2021, no. 1, 1–20, 10.1155/2021/9011168. PMC8531785 34691229

[28] Re R. , Pellegrini N. , Proteggente A. , Pannala A. , Yang M. , and Rice-Evans C. , Antioxidant Activity Applying an Improved ABTS Radical Cation Decolorization Assay, Free Radical Biology and Medicine. (1999) 26, no. 9-10, 1231–1237, 10.1016/S0891-5849(98)00315-3, 2-s2.0-0032982508. 10381194

[29] Benzie I. F. and Strain J. J. , The Ferric Reducing Ability of Plasma (FRAP) as a Measure of “Antioxidant Power”: the FRAP Assay, Analytical Biochemistry. (1996) 239, no. 1, 70–76, 10.1006/abio.1996.0292, 2-s2.0-0030586361. 8660627

[30] Oyaizu M. , Studies on Products of Browning Reaction–Antioxidative Activities of Products of Browning Reaction Prepared from Glucosamine, The Japanese Journal of Nutrition and Dietetics. (1986) 44, no. 6, 307–315, 10.5264/eiyogakuzashi.44.307.

[31] Masalu R. J. , Hosea K. M. , and Malendeja S. , Free Radical Scavenging Activity of Some Fungi Indigenous to Tanzania, Tanzania Journal of Health Research. (2012) 14, 1–8, 10.4314/thrb.v14i1.6, 2-s2.0-84856274251. 26591744

[32] Gul H. , Akbar S. , Gohar S. , and Mazhar F. , Biochemical Analysis and Therapeutic Potential of Extract from Mushroom (*Pleurotus ostreatus*), International Journal of Advanced Computer Research. (2024) 2, no. 1, 84–100.

[33] Lamchouri F. , Settaf A. , Cherrah Y. et al., *In Vitro* Cell-Toxicity of *Peganum harmala* Alkaloids on Cancerous Cell-Lines, Fitoterapia. (2000) 71, no. 1, 50–54, 10.1016/S0367-326X(99)00117-3, 2-s2.0-0033989597. 11449470

[34] Akabli T. , Lamchouri F. , Senhaji S. , and Toufik H. , Molecular Docking, ADME/Tox Prediction, and *in Vitro* Study of the Cell Growth Inhibitory Activity of Five *β*-carboline Alkaloids, Structural Chemistry. (2019) 30, no. 4, 1495–1504, 10.1007/s11224-019-01308-x, 2-s2.0-85062712154.

[35] He M. Q. , Wang M. Q. , Chen Z. H. et al., Potential Benefits and Harms: A Review of Poisonous Mushrooms in the World, Fungal Biology Reviews. (2022) 42, 56–68, 10.1016/j.fbr.2022.06.002.

[36] Zorrilla J. G. and Evidente A. , Structures and Biological Activities of Alkaloids Produced by Mushrooms, a Fungal Subgroup, Biomolecules. (2022) 12, no. 8, 10.3390/biom12081025. PMC9332295 35892335

[37] Patocka J. , Wu R. , Nepovimova E. , Valis M. , Wu W. , and Kuca K. , Chemistry and Toxicology of Major Bioactive Substances in Inocybe Mushrooms, International Journal of Molecular Sciences. (2021) 22, no. 4, 10.3390/ijms22042218. PMC7926736 33672330

[38] Qwarse M. , Marealle A. I. , Machumi F. et al., Exploring the Therapeutic Potential of Wild Edible Mushrooms: Safety Evaluation and Isolation of Antimycobacterial Sterols from *Afrocantharellus platyphyllus* (Heinem.) Tibuhwa, Chemistry Africa. (2023) 7, no. 2, 1–10, 10.1007/s42250-023-00765-6.

[39] Das S. , Study on the Effect of Bioactive Extract of Morel Mushroom Morchella esculenta L Pers, in Mitigating Cancer Chemotherapy drugs-induced Cardiotoxicity, 2025, Amala Cancer Research Centre Thrissur, University of Calicut, https://scholar.uoc.ac.in/bitstreams/7262ee2d-07fc-4441-a36d-ae18288df170/download, Doctoral dissertation.

[40] Monika Thakur M. T. and Lakhanpal T. N. , Qualitative Phytochemical Screening, Total Phenolic Content and in-vitro Antioxidant Activity in Methanolic Extracts of *Morchella esculenta* Fr, *Proceedings of the 8th International Conference on Mushroom Biology and Mushroom Products (ICMBMP8)*, 19th–22nd November, 2014, New Delhi, India, 215–220.

[41] Anand K. , Mandal D. , and Parbhakar P. K. , Phytochemical Profiling, In-Vitro Antioxidant, and Antidiabetic Evaluation of *Morchella Esculenta*: A Comprehensive Investigation, Letters in Applied NanoBioScience. (2024) 14, no. 1, 1–13, 10.33263/LIANBS141.043.

[42] Lachkar N. , Lamchouri F. , and Toufik H. , Ethnopharmacological Survey, Mineral and Chemical Content, *in Vitro* Antioxidant, and Antibacterial Activities of Aqueous and Organic Extracts of *Chamaerops humilis* L. Var. Argentea Andre Leaves, BioMed Research International. (2022) 2022, no. 1, 1–27, 10.1155/2022/1091247. PMC9410792 36033551

[43] Zhang Q. , Wu C. , Sun Y. , Li T. , and Fan G. , Cytoprotective Effect of *Morchella esculenta* Protein Hydrolysate and Its Derivative Against H_2_O_2_-induced Oxidative Stress, Polish Journal of Food and Nutrition Sciences. (2019) 69, no. 3, 255–265, 10.31883/pjfns/110134, 2-s2.0-85072016112.

[44] Bouabid K. , Lamchouri F. , Toufik H. , and Faouzi M. E. A. , Phytochemical Investigation, *in Vitro* and *in Vivo* Antioxidant Properties of Aqueous and Organic Extracts of Toxic Plant: *Atractylis gummifera* L, Journal of Ethnopharmacology. (2020) 253, 1–11, 10.1016/j.jep.2020.112640. 32027998

[45] Senhaji S. , Lamchouri F. , and Toufik H. , Phytochemical Content, Antibacterial and Antioxidant Potential of Endemic Plant *Anabasis Aretioïdes* Coss. & Moq. (Chenopodiaceae), BioMed Research International. (2020) 2020, no. 1, 1–13, 10.1155/2020/6152932. PMC7015181 32076611

[46] Dimitrijevic M. , Jovanovic V. S. , Cvetkovic J. , Mihajilov-Krstev T. , Stojanovic G. , and Mitic V. , Screening of Antioxidant, Antimicrobial and Antiradical Activities of Twelve Selected Serbian Wild Mushrooms, Analytical Methods. (2015) 7, no. 10, 4181–4191, 10.1039/c4ay03011g, 2-s2.0-84929380108.

[47] Badshah S. L. , Riaz A. , Muhammad A. et al., Isolation, Characterization, and Medicinal Potential of Polysaccharides of *Morchella esculenta* , Molecules. (2021) 26, no. 5, 1–12, 10.3390/molecules26051459. PMC7962536 33800212

[48] Hira F. A. , Islam A. , Mitra K. et al., Comparative Analysis of Phytochemicals and Antioxidant Characterization Among Different Parts of Catharanthus Roseus: *in Vitro* and *in Silico* Investigation, Biochemistry Research International. (2024) 2024, no. 1, 1–22, 10.1155/2024/1904029. PMC11519068 39474335

[49] Jafri L. , Saleem S. , Ul-Haq I. , Ullah N. , and Mirza B. , *In Vitro Assessment of Antioxidant Potential and Determination of Polyphenolic Compounds of Hedera nepalensis K. Kochn Vitro* Assessment of Antioxidant Potential and Determination of Polyphenolic Compounds of *Hedera nepalensis* K. Koch, Arabian Journal of Chemistry. (2014) 10, 3699–3706, 10.1016/j.arabjc.2014.05.002, 2-s2.0-84902906064.

[50] Ribeiro A. L. , Antunes M. V. G. , Oliveira C. F. et al., Phytochemical Evaluation and Antioxidant Potential of *Echinodorus macrophyllus* Extracts, Brazilian Journal of Pharmaceutical Sciences. (2025) 61, 1–14, 10.1590/s2175-97902025e23933.

[51] Stoclet J. C. and Schini-Kerth V. , Dietary Flavonoids and Human Health, Annales Pharmaceutiques Françaises. (2011) 69, no. 2, 78–90, 10.1016/j.pharma.2010.11.004, 2-s2.0-79953029069. 21440100

[52] Tumilaar S. G. , Hardianto A. , Dohi H. , and Kurnia D. , A Comprehensive Review of Free Radicals, Oxidative Stress, and Antioxidants: Overview, Clinical Applications, Global Perspectives, Future Directions, and Mechanisms of Antioxidant Activity of Flavonoid Compounds, Journal of Chemistry. (2024) 2024, no. 1, 1–21, 10.1155/2024/5594386.

[53] Dang Lelamurni A. R. , Mohd Fadzil N. H. , Jamaluddin A. , Abd Rashid N. Y. , Sani N. A. , and Abdul Manan M. , Effects of Different Extracting Conditions on Anti-tyrosinase and Antioxidant Activities of *Schizophyllum commune* Fruit Bodies, Biocatalysis and Agricultural Biotechnology. (2019) 19, 1–6, 10.1016/j.bcab.2019.101116, 2-s2.0-85063966739.

[54] Gursoy N. , Sarikurkcu C. , Cengiz M. , and Solak M. H. , Antioxidant Activities, Metal Contents, Total Phenolics and Flavonoids of Seven Morchella Species, Food and Chemical Toxicology. (2009) 47, no. 9, 2381–2388, 10.1016/j.fct.2009.06.032, 2-s2.0-67949097251. 19563856

[55] Huyut Z. , Beydemir Ş. , and Gülçin İ. , Antioxidant and Antiradical Properties of Selected Flavonoids and Phenolic Compounds, Biochemistry research international. (2017) 2017, no. 1, 1–10, 10.1155/2017/7616791, 2-s2.0-85042227487. PMC5660747 29158919

[56] Luciano Braguini W. , Valendolf Pires N. , and Bianchin Alves B. , Phytochemical Analysis, Antioxidant Properties and Brine Shrimp Lethality of Unripe Fruits of *Solanum viarum* , Journal of Young Pharmacists. (2018) 10, no. 2, 159–163, 10.5530/jyp.2018.10.36, 2-s2.0-85045852393.

[57] Hassan M. , Al-Ragib A. , Tanvir Hossai Md. , Sazib S. M. , and Islam M. Z. , Evaluation of Antioxidant Activity and Brine Shrimp Lethality Bioassay of *Randia dumetorum* Stem Extract, North American Academic Research. (2020) 3, no. 01, 1–14, 10.5281/zenodo.3611649.

